# Supplementary figures and images for: Comparing COVID-19 physical distancing policies: results from a physical distancing intensity coding framework for Botswana, India, Jamaica, Mozambique, Namibia, Ukraine, and the United States
Source: Global Health. 2021 Oct 23;17:124. doi: 10.1186/s12992-021-00770-9 (PMC8541811; doi:10.1186/s12992-021-00770-9)

# Botswana

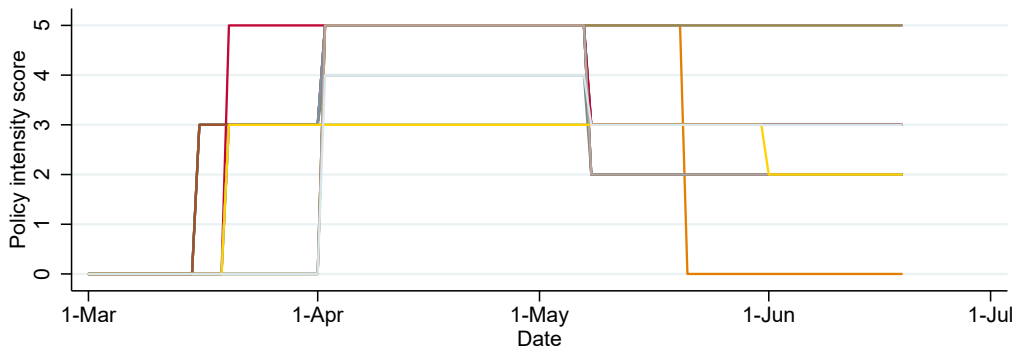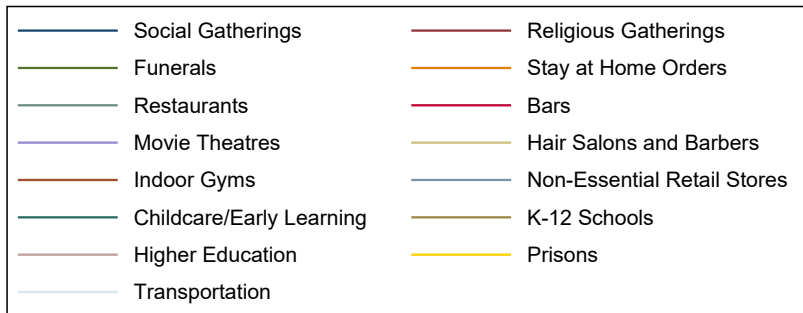

# India

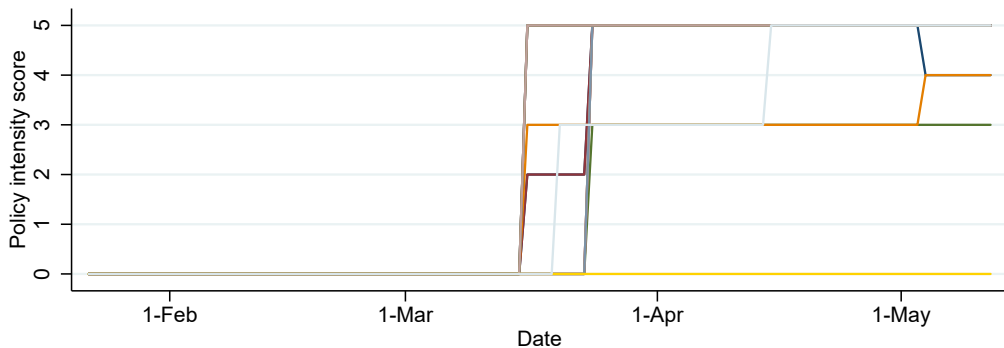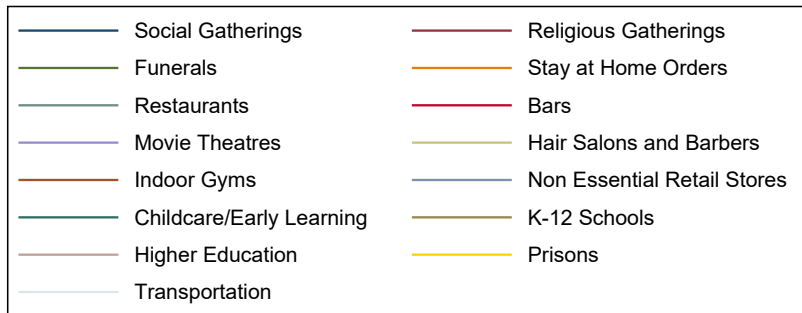

# Jamaica

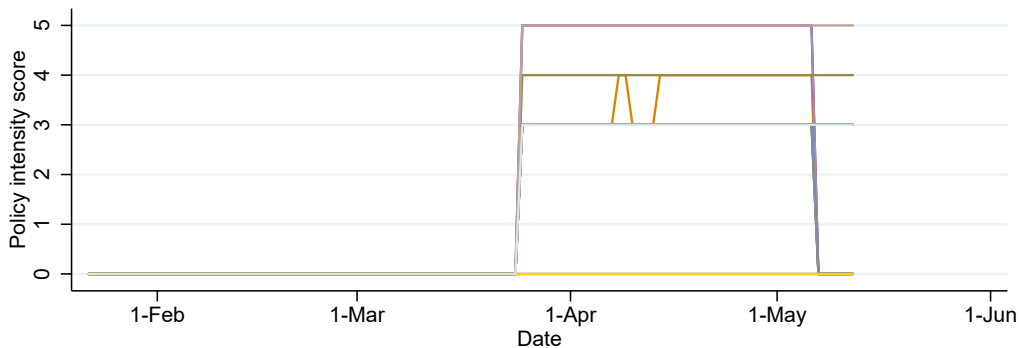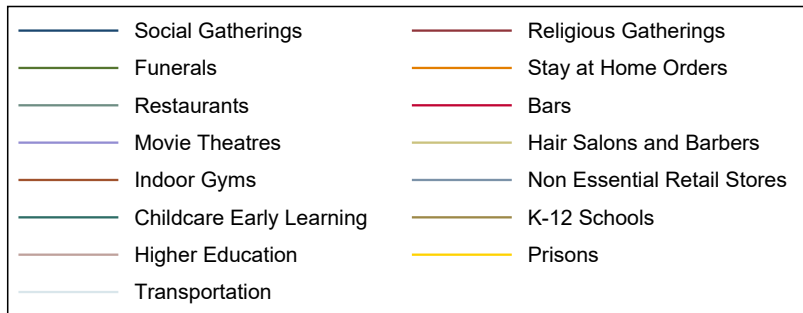

# Mozambique

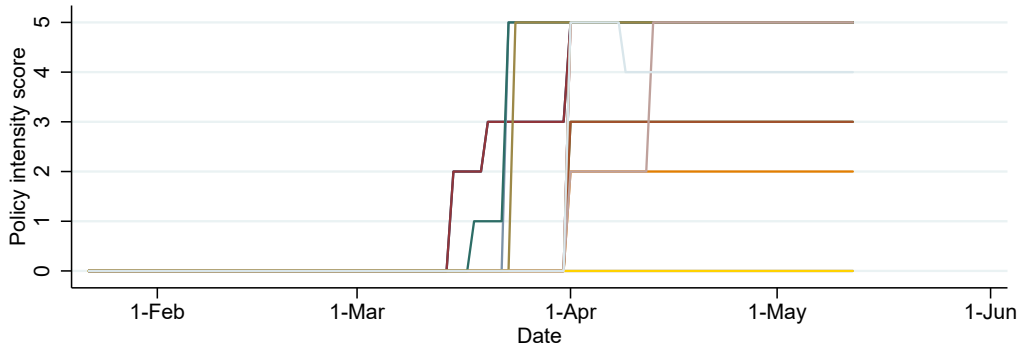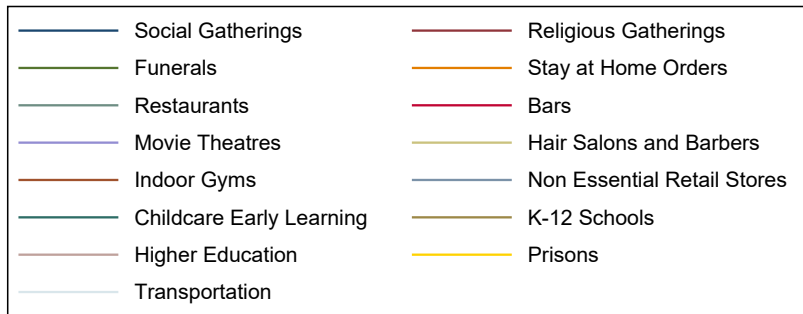

# Namibia

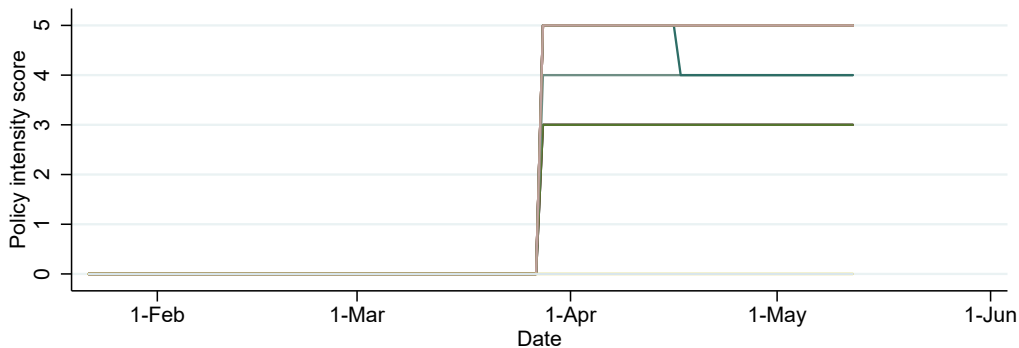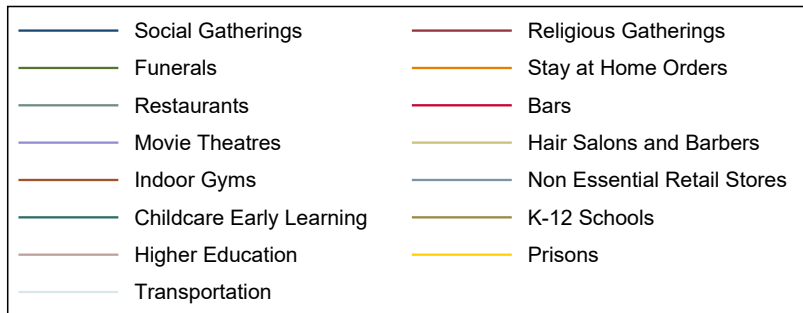

# Ukraine

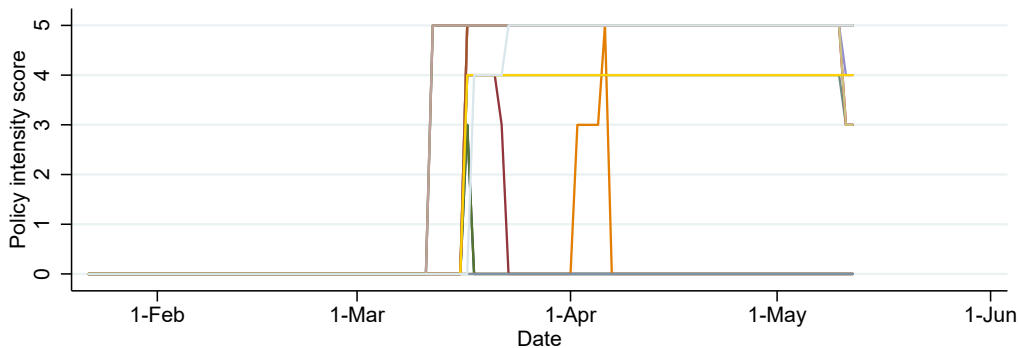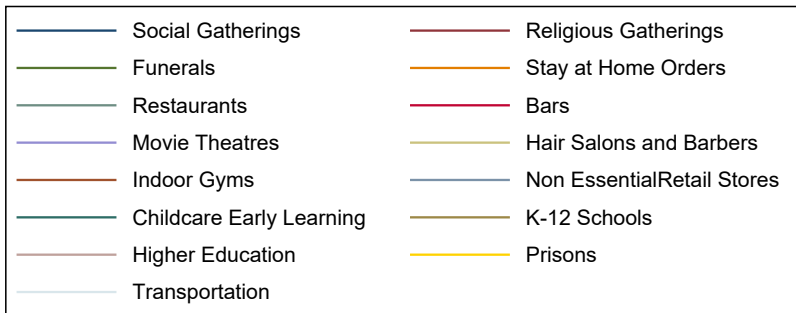

Supplement: Supplementary file 1 — Additional file 1 [file 12992_2021_770_MOESM1_ESM.pdf]
